# Expression of N-cadherin proteins in myocardial hypertrophy in rats

**DOI:** 10.3892/etm.2013.1431

**Published:** 2013-11-28

**Authors:** LINGMIN MU, CHANGQIN JING, ZHIKUN GUO

**Affiliations:** 1 Morphological Laboratory, Xinxiang Medical University, Xinxiang, Henan 453003, P.R. China; 2 Life Science and Technology Department, Xinxiang Medical University, Xinxiang, Henan 453003, P.R. China; 3 Key Open Laboratory for Tissue Regeneration in Henan Province, Xinxiang Medical University, Xinxiang, Henan 453003, P.R. China

**Keywords:** immunohistochemistry, immunofluorescence, myocardial hypertrophy, N-cadherin, reverse transcription polymerase chain reaction

## Abstract

The aim of the present study was to examine the expression of N-cadherin in the myocardial tissues of isoproterenol-induced myocardial hypertrophy in rats. In addition, the present study provided morphological data to investigate the signal transduction mechanisms of myocardial hypertrophy and reverse myocardial hypertrophy. A myocardial hypertrophy model was established by subcutaneously injecting isoprenaline into healthy adult Sprague-Dawley rats. The myocardial tissue was collected, embedded in conventional paraffin, sectioned and stained with hematoxylin and the pathological changes were observed. The expression and distribution of N-cadherin were detected by immunohistochemistry (IHC) and the changes in mRNA expression of N-cadherin in the myocardial tissues of rats were detected by reverse transcription polymerase chain reaction. Image analysis software was used to quantitatively analyze the expression of N-cadherin. The IHC and immunofluorescence results showed that there was no statistically significant difference between the experimental and control groups in the positive expression of N-cadherin. Furthermore, mRNA expression of N-cadherin, in the myocardial tissues of rats, was consistent with the IHC and immunofluorescence results. Thus, N-cadherin may have a significant function in the occurrence and development of myocardial hypertrophy.

## Introduction

Myocardial hypertrophy is a normal adjustment in response to wall stress resulting from an increased cardiac load (1,2). The predominant characteristics of myocardial hypertrophy include an increased cell volume, enhanced protein synthesis and an upregulation of the expression of embryonic genes. The predominant characteristics of the histopathology include an expanded cell space, increased intercellular muscle fibers as well as myocardial fibrosis and dysfunction.

The increasing incidence and prevalence of heart failure (HF) has been identified as one of the predominant causes of morbidity and mortality in the elderly (3,4). The pathogenesis of HF is complex and often associated with cardiac remodeling, which involves cardiac myocyte hypertrophy, re-expression of the fetal program and phenotypic changes within the extracellular matrix (3–5). Myocardial hypertrophy may occur as an adaptive response to a pressure or volume overload as a result of decreasing wall stress, whereas chronic left ventricle hypertrophy is strongly associated with chronic HF and fatalities. Moreover, chronic left ventricle hypertrophy is considered a maladaptive process, thereby inducing a fetal gene program and pro-hypertrophic signaling pathways (6–8). In an adult heart, hypertrophic growth results from signals that are stimulated at the cell surface and subsequently transmitted via receptors or channels, which activate intracellular signaling cascades and affect nuclear cues, thereby altering gene expression (9,10). The molecular machinery that directs mechanical sensing in cardiac myocytes is partially understood. In certain cases, the cell surface adhesion receptors, termed N-cadherins, are significant detectors of mechanical load.

Cadherins are Ca^2+^-dependent transmembrane proteins, which form a large family of cell-to-cell adhesion molecules. At present, >100 members of the cadherin superfamily have been identified in invertebrates and vertebrates, with the majority predominantly comprising extracellular, transmembrane and cytoplasmic domains (11,12). In addition, the optimally characterized cadherins were further classified into epithelial (E), neural (N) and placental (P) cadherins based on the tissue distribution that was initially identified. The cadherin gene was identified by Yagi and Takeichi (13) 30 years previously and the function of cadherins has recently been demonstrated. This function is not limited to mechanical adhesion, but is key in cellular localization, proliferation and differentiation through the cadherin-catenin-cytoskeleton complex, which transforms extracellular stimuli into intracellular signals (12,13). It has been demonstrated that the cardiomyocytes can be mechanically joined through N-cadherin-mediated cell adhesion, which simultaneously provides the anchor point for the cytoskeleton. This mechanical cell adhesion maintains the structural integrity and polarity of the tissues in the adult organism (14,15). Clinical and experimental studies have been conducted to explore the expression and function of N-cadherin in tumors and cancer cells. However, few studies have focused on the expression and function of N-cadherin in cardiomyocytes following a myocardial hypertrophy-induced increase in the intercellular space.

In the present study, the N-cadherin expression was investigated based on a myocardial hypertrophy model established by subcutaneously injecting isoprenaline (ISO) into rats. The expression and distribution of N-cadherin in the myocardial tissue were observed to provide morphological data, in addition to investigating the signal transduction mechanisms of myocardial and reverse myocardial hypertrophy.

## Materials and methods

### Establishment of a rat model of myocardial hypertrophy

The rat model of myocardial hypertrophy was established as previously described (16). Twenty healthy, adult Sprague-Dawley rats (10 males and 10 females; weight, 200±20 g) were provided by the Experimental Animal Center of Xinxiang Medical University (Henan, China). The rats were randomly divided in two groups with 10 rats per group. The rats in the experimental group were subcutaneously injected with 8 mg/kg/day ISO (Shanghai Harvest Pharmaceutical Co. Ltd., Shanghai, China) twice a day for five consecutive days and observed for 48 h. The rats in the control group were subcutaneously injected with 2 ml/kg/day of physiological saline according to the same procedure as the experimental group. This study was conducted with approval from the Ethics Committee of Xinxiang Medical University (Xinxiang, China).

### Determination of the myocardial hypertrophy index

The body weight (BW) of the rats in the two groups was measured immediately following the observation period. The rats were narcotized via an intraperitoneal injection of 20% ethyl carbamate solution (750 mg/kg) and supination and fixation were performed. Regular shearing and disinfection were conducted and the chests of the rats were opened to remove the heart. The heart was subsequently washed with pre-cooled normal saline until the flushing fluid was not red and clean filter paper was used to absorb the moisture. The tissues and blood vessels surrounding the heart were cut and the heart weight (HW) was measured. The left and right atria, along the coronary artery groove, and the right ventricular free wall, along the interventricular groove, were removed and the left ventricular weight (LVW) was measured. HW/BW and LVW/BW were calculated to determine the extent of myocardial hypertrophy. Two myocardia (each weighing ~0.3 g) from the left ventricle were obtained from the two groups of rats. One myocardium was immediately placed in 4% neutral formaldehyde stationary liquid, embedded with conventional paraffin, sectioned and subjected to immunohistochemistry (IHC), hematoxylin and eosin (H&E) staining and immunofluorescence. The second myocardium was placed in liquid nitrogen.

### H&E staining

The myocardial tissue was embedded in conventional paraffin and sectioned. Following the standard process of H&E staining (17), the specimens were observed under a light microscope (Nikon E400; Shanghai Weihan Optoelectronic Techonogy Company; Shanghai, China) and the ratio of myocardial cells to capillaries, the diameter of cardiomyocytes, cell density, capillary density, intracellular substance and intercellular space were examined to evaluate the extent of myocardial hypertrophy.

### IHC staining method

The myocardial tissue was embedded in conventional paraffin and sectioned using an SP-9001 IHC staining kit in accordance with the manufacturer’s instructions. Primary antibodies of N-cadherin (1:100; Wuhan Boster Biological Technology Ltd., Wuhan, China) were incubated overnight at 4°C and subsequently incubated at 37°C for 30 min in biotin-labeled IgG and streptavidin-biotin complex liquid. The specimens were stained with 3,3′-diaminobenzidine and re-dyed with hematoxylin. Phosphate-buffered saline was used for the negative control specimens. The specimens were observed under a light microscope and photographed. Brown reaction granules observed in the cells indicated a positive result. Six myocardial specimens were obtained from each group of IHC results, five sections were selected per specimen and four views were obtained from the selected sections, which showed uniform myocardial tissue distribution and dyeing. The results were quantitatively analyzed using a Motic BA400 pathological graphic analysis system (Motic China Group Company, Guangdong, China; magnification, ×400). The positive expression area and the average optical density were considered to be key indicators of N-cadherin.

### Immunofluorescence method

The myocardial tissue was embedded in conventional paraffin and sectioned. Following incubation with 3% H_2_O_2_, antigen microwave repair and serum were incubated at 37°C for 30 min. The specimens were then incubated with rabbit anti-rat N-cadherin polyclonal antibody at 37°C for 1 h. The specimens were subsequently incubated with fluorescein isothiocyanate-labeled goat anti-rabbit IgG at 37°C for 1 h and nuclear staining was conducted using 4′,6-diamidino-2-phenylindole (DAPI) for 5–8 min. The specimens were then sealed with glycerol and observed under an FV1000 confocal microscope (Olympus; Shanghai Weihan Optoelectronic Techonogy Company).

### Reverse transcription polymerase chain reaction (RT-PCR)

The heart tissue was removed from the liquid nitrogen and total RNA was extracted using an AxyPrep total RNA preparation kit (Zhengzhou Baosai Biology Technology Company, Zhengzhou, China) in accordance with the manufacturer’s instructions. Ultraviolet (UV) absorbance at 260 and 280 nm was determined using a UVmini-1240 UV spectrophotometer (Zhengzhou Baosai Biology Technology Company, Zhengzhou China) and the RNA integrity was detected by 1% agarose gel electrophoresis. The N-cadherin primer sequences were designed using Primer 5 primer design software (Table I).

The primers were synthesized by the GeneGenius Agar imaging analysis system (Syngene, Frederick, MD, USA), purified via the PAGE method, dissolved in RNase-free water to 100 μmol/l and preserved at −70°C for later use. First-strand cDNA was synthesized using an M-MLV reverse transcription kit kit (Zhengzhou Baosai Biology Technology Company) according to the manufacturer’s instructions. The N-cadherin was amplified and the amplification reaction was recorded in a single system using β-actin as an internal reference. The reaction conditions used were initial denaturation at 95°C for 5 min, loop degeneration at 94°C for 30 sec, renaturation at 55°C for 30 sec and extension at 72°C for 10 min. The PCR products were electrophoresed in 1.5% agarose gel (containing ethidium bromide with a 0.5-mg/ml final concentration). The GeneGenius Agar imaging analysis system (Syngene, Frederick, MD, USA) and Gelworks 10 software were used to scan and record the results. The gray value ratios of target fragment/β-actin were calculated by semi-quantitative analysis.

### Statistical analysis

Data were presented as mean ± standard deviation. The gray value ratios of N-cadherin/β-actin were calculated and analyzed in pairs using SPSS 14.0 (SPSS Inc., Chicago, IL, USA) via variance analysis.

## Results

### Determination of the myocardial hypertrophy index

The experimental group exhibited an increase in HW/BW and LVW/BW based on the myocardial hypertrophy index analysis, compared with the control group (P<0.05 and P<0.01; Table II).

### Myocardial histopathology results

**Figure 1:**
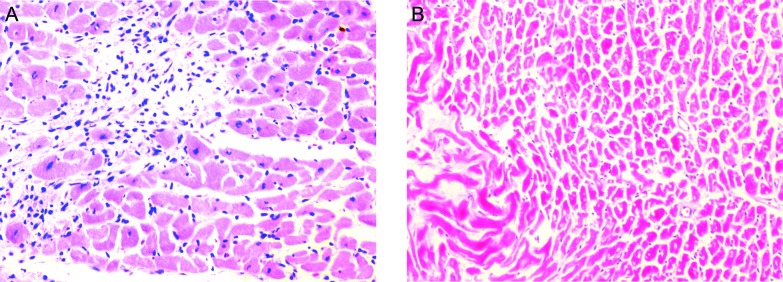
Morphological change of the rat myocardial tissue following isoproterenol treatment by H&E staining. (A) Experimental group. (B) Control group. Rats in the experimental group were injected with isoprenaline, while rats in the control group were injected with physiological saline. Myocardial tissues were collected, embedded in conventional paraffin, sectioned and stained with hematoxylin. Magnification, ×400.

Even staining of the cardiomyocytes was observed in the control group specimens in addition to a neat arrangement with clearly visible stripes. However, the cardiomyocytes in the experimental group exhibited varying sizes and stained unevenly. Furthermore, increases in the cell diameter, intercellular space and nuclear size were observed, however, the density of the cell and blood capillaries decreased, compared with the cardiomyocytes in the control group. Nuclear vacuolation was also observed in the experimental group (Fig. 1).

### IHC results

**Figure 2:**
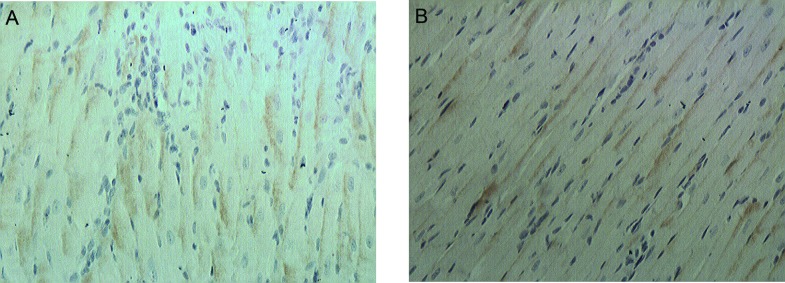
N-cadherin expression in the myocardial tissue by immunohistochemical method. (A) Control group. (B) Experimental group. Magnification, ×400.

The expression of N-cadherin in the intercellular space of the myocardial cells was positive yet weak, and understained brown stripes were observed in the control and experimental groups (Fig. 2). No statistically significant difference was identified between the control and experimental groups in the positively expressed areas and the average optical density of N-cadherin (Table III).

### Immunofluorescence results

**Figure 3:**
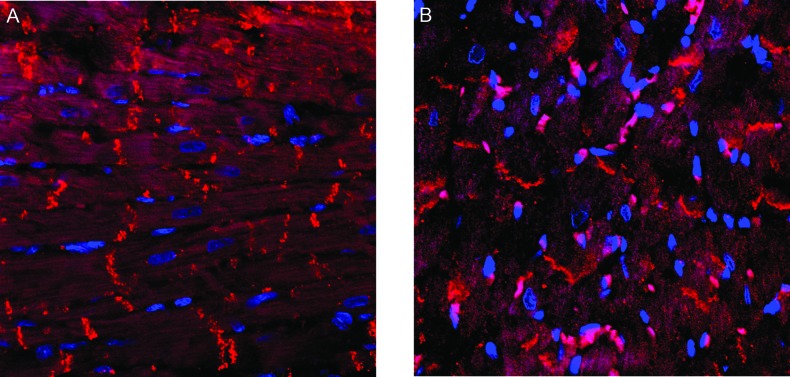
N-cadherin expression in the myocardial tissue by immunofluorescence method. (A) Control group. (B) Experimental group. Magnification, ×400.

Following staining with Rabbit anti-rat N cadherin (Beijing Zhongshan Jinqiao Biological Technology Company, Beijing, China) and DAPI, the N-cadherin protein was labeled red and the nucleus of the myocardial cells was stained blue. The results showed that N-cadherin was predominantly expressed in the intercalated disk of the myocardium, with a small number of cells showing a positive expression and very few cells showing a strongly positive expression (Fig. 3).

### RT-PCR amplification of N-cadherin

**Figure 4:**
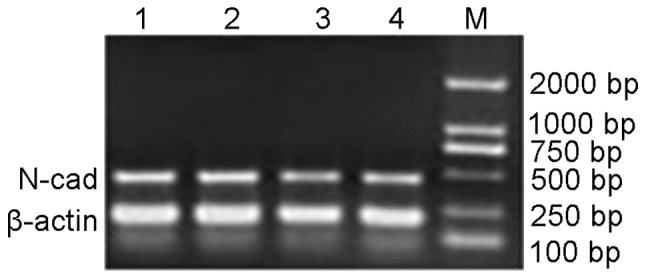
Reverse transcription polymerase chain reaction; products of N-cadherin. M, Marker; 1–2, experimental group; 3–4, control group.

Unique bands were observed at 400–500 bp in the experimental and control groups (Fig. 4). The gray value ratios of N-cadherin/β-actin were calculated and analyzed in pairs using SPSS 14.0 (SPSS Inc., Chicago, IL, USA) via variance analysis. No statistically significant differences were found between the control and experimental groups with regard to the abundance of N-cadherin mRNA.

## Discussion

Myocardial hypertrophy is a normal adjustment in response to wall stress resulting from an increased cardiac load (1,2). The pathogenesis of myocardial hypertrophy has been a controversial topic in the study of cardiovascular diseases (18,19). However, no clear conclusions have been determined.

Cadherins are a superfamily of adhesion molecules that mediate Ca^2+^-dependent cell-to-cell adhesion in all of the solid tissues of an organism. The recent increase in genomic sequencing of the DNA of various animals has revealed novel information regarding the diversity of the cadherin superfamily (11–15). In humans, >80 members of the cadherin superfamily have been sequenced (20). The analysis of proteins and cDNA sequences has revealed that different cadherins are comparable in their overall primary structure; their mature forms consist of 723–748 amino acids and have a single transmembrane domain, which divides the molecules into the N-terminal extracellular domain and the C-terminal cytoplasmic domain. The cytoplasmic domain of cadherins is associated with cytoplasmic protein catenins, which serve as intermediate links between the cadherins and the actin filaments (21,22).

Currently, the analysis of cadherins emphasizes the similarities between embryonic and neural morphogenesis. Cadherins have emerged as the predominant group of cell-to-cell adhesion molecules involved in embryonic morphogenesis, determining cell and tissue architecture in addition to controlling the dynamic changes in cell shape and position (13,23,24). However, the role of N-cadherin in myocardial hypertrophy remains to be determined. Therefore, in the present study, an ISO-induced myocardial hypertrophy model was established. IHC, immunofluoresence and RT-PCR were performed to investigate the changes of distribution, protein expression and the abundance of N-cadherin mRNA.

In conclusion, the IHC and immunofluorescence results indicated that there was no statistically significant difference between the experimental and control groups in the positive expression of N-cadherin. Furthermore, mRNA expression of N-cadherin within the myocardial tissues of rats was consistent with the IHC and immunofluorescence results. Based on these results, it was hypothesized that the N-cadherin adhesion molecule may enhance cell-to-cell contact and provide a sufficient membrane connection area between cells, which enables the heart to maintain its physical structure and mechanical function. N-cadherin may, therefore, be essential to the survival of the heart.

## Figures and Tables

**Table I:** Oligonucleotide primers of N-cadherin and β-actin.

Gene	Sense	Antisense	Product (bp)
N-cadherin	tgttgctgcagaaaaccaag	tttcacaagtctcggcctct	460
β-actin	cacccgcgagtacaaccttc	cccatacccaccatcacacc	206

**Table II:** Index of the rat model of cardiac hypertrophy (mean ± standard deviation).

Groups	No. of rats	HW/BW (mg/g)	LVW/BW (mg/g)
Control	10	6.150±0.619^a^	4.309±0.482^b^
Experimental	10	4.388±0.308	2.081±0.196

a P<0.05 and

b P<0.01 indicates a response that was significantly different to the control group.

HW, heart weight; BW, body weight; LVW, left ventricular weight.

**Table III:** Comparison of the average area and brightness of N-cadherin expression in the myocardium (mean ± standard deviation).

Groups	No. of rats	Average brightness	Average area
Experimental	10	0.51±0.02^a^	127±5^a^
Control	10	0.48±0.02	122±6

a P<0.01 indicates a response that was significantly different to the control group.
